# Trial of Labor After Cesarean and Vaginal Birth After Cesarean: A Systematic Review and Meta-Analysis of Maternal and Neonatal Outcomes

**DOI:** 10.3390/medicina62071286

**Published:** 2026-07-03

**Authors:** Elitsa Gyokova, Eleonora Hristova-Atanasova, Angel Yordanov, Eva Tsoneva, Zlatko Kirovakov

**Affiliations:** 1 Department of Obsterics and Gynecology, Faculty of Medicine, Medical University—Pleven, 5800 Pleven, Bulgaria; egyokova@yahoo.com; 2 Clinic of Obstetrics and Gynecology, University Hospital “Saint Marina’’, 5800 Pleven, Bulgaria; 3 Department of Social Medicine and Public Health, Faculty of Public Health, Medical University—Plovdiv, 4002 Plovdiv, Bulgaria; 4 Department of Gynaecological Oncology, Medical University—Pleven, 5800 Pleven, Bulgaria; 5 Department of Reproductive Medicine, Specialized Hospital for Active Treatment of Obstetrics and Gynaecology Dr Shterev, 1330 Sofia, Bulgaria; dretsoneva@gmail.com; 6 Faculty of Public Health and Health Care, Burgas State University “Prof. D-R Assen Zlatarov”, 8000 Burgas, Bulgaria; kirovakov@yahoo.com

**Keywords:** trial of labor after cesarean (TOLAC), elective repeat cesarean delivery (ERCD), uterine rupture, postpartum hemorrhage, maternal outcomes, maternal morbidity

## Abstract

*Background and Objectives*: The rising rate of cesarean delivery worldwide has led to an increasing number of women considering their options for birth after a previous cesarean section. Vaginal birth after cesarean (VBAC) offers potential maternal benefits but remains associated with concerns regarding maternal and neonatal safety. This systematic review and meta-analysis aimed to evaluate VBAC success rates and compare maternal and neonatal outcomes associated with trial of labor after cesarean (TOLAC) and elective repeat cesarean delivery (ERCD). *Materials and Methods*: A systematic review and meta-analysis was conducted according to PRISMA guidelines. PubMed/MEDLINE, Embase, and CENTRAL were searched for studies published between January 2010 and January 2026. Pooled proportions were calculated for VBAC success rates, while pooled risk ratios (RRs) with 95% confidence intervals (CIs) were used to evaluate comparative outcomes when sufficient data were available. *Results*: Thirty-one studies, including one randomized controlled trial and thirty observational studies, were included. The overall VBAC success rate was 67.7% (95% CI 61.8–73.6%), although substantial heterogeneity was observed across studies (I^2^ = 98.7%). No evidence of significant small-study effects was identified. Women undergoing TOLAC had a higher, although imprecisely estimated, risk of uterine rupture compared with those undergoing ERCD (RR 6.86, 95% CI 0.72–65.07). No statistically significant differences were found for maternal transfusion (RR 2.08, 95% CI 0.43–10.14) or postpartum hemorrhage (RR 1.47, 95% CI 0.04–55.03). Neonatal outcomes were reported inconsistently and could not be reliably pooled. *Conclusions*: VBAC may be an appropriate option for carefully selected women after individualized assessment of the likelihood of successful vaginal birth and the potential risks associated with TOLAC. However, the available evidence is based predominantly on observational studies, and the certainty of evidence for most comparative outcomes remains low or very low. These findings support individualized counselling and shared decision-making while highlighting the need for larger, well-designed comparative studies with standardized outcome reporting.

## 1. Introduction

In the past few decades, there has been a significant increase in the incidence of cesarean section (CS) deliveries, with projections of up to a third of global deliveries being done through the procedure by 2030 [1]. According to the most recent available statistics from 154 countries covering 94.5% of world live births, the global cesarean section births stand at 21.2 percent with averages ranging from 5% in sub-Saharan Africa to 42.8% in Latin America and the Caribbean, 25.7% in Europe, 23.1% in Asia, and a marked overall increase in all regions since 1990 [2,3,4,5]. Every subsequent pregnancy for the millions of women who have had a previous cesarean delivery requires them to make the crucial decision of whether to arrange for an elective repeat cesarean delivery (ERCD) or try a trial of labor after cesarean (TOLAC) to achieve a successful vaginal birth after cesarean (VBAC) [6,7,8]. In the early 2000s, there was an abrupt reduction in VBAC procedures in many high-income nations due to concerns about the danger of uterine rupture and related perinatal morbidity, a trend which was further exacerbated by extensive research and medico-legal pressures [9,10,11]. Still, the overall cesarean rate has continued to climb, driven largely by repeat procedures, which has created a persistent tension in obstetrics involving the iatrogenic risks of major abdominal surgery with each repeat cesarean versus the potentially catastrophic, though rare, risk of uterine rupture during TOLAC [12,13]. An understanding of the evolving landscape of VBAC on the basis of core risk-benefit analysis and predictive factors for success can be important in addressing this issue which is becoming a global concern in obstetrics. In this regard, it makes sense that reducing a chain of repeat procedures is part of the current efforts to avoid needless primary cesarean sections.

Elective repeat cesarean delivery (ERCD), while predictable and avoiding the specific risks of labor, is associated with higher risks of maternal morbidity compared with successful VBAC, including increased rates of surgical injury, infection, thromboembolism, abnormal placentation (placenta previa and accreta spectrum) in future pregnancies, and longer recovery times [14,15]. In contrast, a successful VBAC provides the widely recognized advantages associated with vaginal birth, including reduced complications, shorter hospital stays, and increased maternal satisfaction [16]. The primary risk linked to TOLAC is uterine rupture, a rare but catastrophic event that occurs in about 0.5–0.9% of cases and can lead to hysterectomy, perinatal asphyxia, and neonatal mortality [17,18]. Over the last two decades, researchers have greatly improved our capacity to predict VBAC success and failure, and individual characteristics have been highly associated with outcomes. A prior vaginal delivery, particularly a prior VBAC, is the strongest predictor of success, with rates exceeding 85% [19], and other favorable factors include spontaneous commencement of labor, a non-recurrent rationale for the original cesarean (e.g., breech), and a positive cervical status [20]. On the other hand, factors associated with reduced success (often quoted at <60–70%) include maternal obesity, advanced maternal age, gestational age over 40 weeks, and the need for labor induction or augmentation, especially with prostaglandins [21,22]. Therefore, the accurate identification of those patients for whom the likelihood of a successful VBAC outweighs the accompanying risks is crucial to clinical care and patient counseling.

To advance beyond isolated parameters, multivariable prediction models have been extensively adopted, most notably the Maternal-Fetal Medicine Units (MFMU) VBAC calculator, which integrates several maternal and obstetric features to produce an individualized prediction of success [23], and has become integral to shared decision-making, allowing clinicians and patients to contextualize risks within a personalized framework. Despite these developments, there are still a lot of uncertainties and gaps in the evidence, which makes it necessary to continuously undertake high-caliber research using systematic reviews and meta-analyses with key areas of concern and contention being the safety of TOLAC for individuals with two prior cesareans, multiple gestation, or an unknown uterine scar type. Also, the largest proportion of existing research on the topic originates from high-resource settings with immediate surgical capabilities and, therefore, the feasibility and safety of VBAC in low- and middle-income countries, where emergency obstetric care may be limited, represent a critical and understudied frontier [24]. Accordingly, more research is needed in the areas of long-term pelvic floor health, future fertility, and the psychological effects of birth mode preference against the outcomes [25]. To this end, the present review aims to provide a comprehensive synthesis of contemporary evidence regarding VBAC success rates and maternal and neonatal outcomes associated with TOLAC. Particular emphasis is placed on comparative outcomes between TOLAC and elective repeat cesarean delivery (ERCD), where available, to support evidence-based counselling, individualized risk assessment, and shared decision-making for women with a previous cesarean delivery.

## 2. Materials and Methods

This systematic review and meta-analysis were conducted and reported in accordance with the Preferred Reporting Items for Systematic Reviews and Meta-Analyses (PRISMA) guidelines (Table S3). The study protocol was registered prospectively with the International Prospective Register of Systematic Reviews (PROSPERO; ID: CRD420261384006).

### 2.1. Search Strategy and Information Sources

A comprehensive literature search was performed to identify all relevant studies published from 1 January 2010 to 15 January 2026. The search was conducted across three major electronic databases: PubMed/MEDLINE, Embase (via Ovid), and CENTRAL. The last literature search was conducted on 15 January 2026. The Medical Subject Headings (MeSH) terms and keywords related to “vaginal birth after cesarean,” “trial of labor,” “repeat cesarean section,” and associated outcomes were used. Studies were selected based on predefined inclusion and exclusion criteria developed according to the PICO framework. No language restrictions were applied initially; non-English articles were translated for full-text assessment. The complete electronic search strategies for each database are provided in Supplementary Table S2.

### 2.2. Eligibility Criteria

Studies were selected based on the following criteria:

***Inclusion Criteria:***

1. **Population:** Pregnant individuals with one or more previous low-transverse cesarean deliveries. Selected cohorts of women undergoing vaginal birth after two previous cesarean deliveries (VBA2C) were considered eligible provided that no classical, T-shaped, or unknown uterine scars were included, or that data for women with low-transverse scars were reported separately.
2. **Intervention/Exposure:** Planned trial of labor after cesarean (TOLAC) with the intention of achieving VBAC.
3. **Comparator:** Elective repeat cesarean delivery (ERCD), where available.
4. **Outcomes:** Reported at least one primary or secondary outcome of interest.

In addition, protocol publications were retained only when they provided methodological information relevant to a subsequently completed randomized controlled trial included in the review. Protocol publications were not considered independent sources of outcome data and did not contribute to the quantitative synthesis.

Studies with different objectives and designs contributed differently to the review. Comparative meta-analyses were restricted to studies providing directly extractable TOLAC versus ERCD outcome data. Studies without comparator groups, including descriptive cohorts and selected case-series studies meeting the predefined sample-size criterion, contributed only to pooled VBAC success estimates and narrative synthesis. Prediction studies and studies evaluating determinants of successful VBAC were included to characterize factors associated with VBAC success but did not contribute to comparative outcome meta-analyses.

5. **Publication date:** Studies published from 2010 onward.

***Exclusion Criteria:***

1. Studies including individuals with prior classical, T-shaped, or unknown uterine incisions unless data for the low-transverse cohort were reported separately.
2. Case reports, case series (<50 participants), editorials, commentaries, and narrative reviews without original data.
3. Studies with overlapping patient populations.
4. Conference abstracts for which the full text could not be retrieved.

### 2.3. Study Selection Process

**Figure 1:**
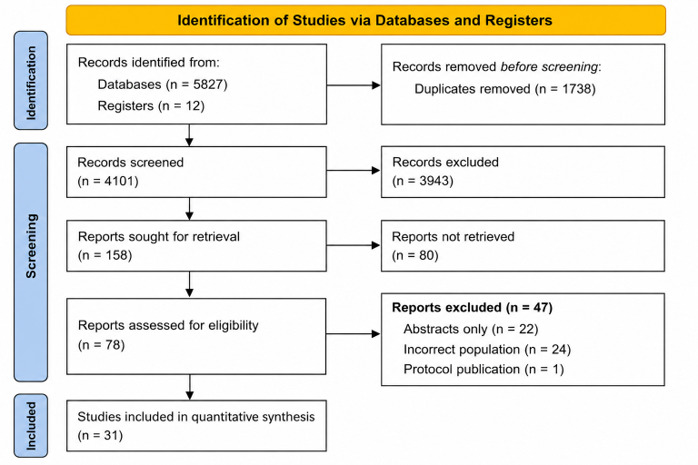
PRISMA flow diagram.

All identified records were imported into systematic review software for management. After removal of duplicates, two independent reviewers screened titles and abstracts against the eligibility criteria. The full texts of potentially relevant articles were then retrieved and assessed independently. The study selection process is detailed in the PRISMA flow diagram (Figure 1). Detailed reasons for full-text exclusion, including incorrect study population, lack of extractable outcome data, overlapping populations, ineligible study design, and inaccessible full-text publications, are provided in Supplementary Table S2.

### 2.4. Data Extraction and Management

After selecting relevant and appropriate studies for evaluation, useful information about the topic and variables of interest was extracted using a pre-developed standardized data extraction form to ensure data collection uniformity and completeness. The extracted data for each included article were study author and identification, design and methodology, population characteristics, outcomes and key findings. Each included study’s data was retrieved separately by two reviewers. Extracted variables included study design, country, sample size, TOLAC/VBAC group size, ERCD group size where available, VBAC success rate, maternal outcomes, neonatal outcomes, and reported predictors of successful VBAC.

### 2.5. Risk of Bias (Quality) Assessment

The methodological quality and potential risk of bias of the included studies were independently assessed by two reviewers. The quality of observational studies was evaluated using the Newcastle–Ottawa Scale (NOS), which assesses participant selection, comparability between study groups, and outcome assessment. Studies with NOS scores of 7–9 were considered high quality and at low risk of bias, scores of 5–6 indicated moderate quality, and scores of 4 or below were considered to represent a high risk of bias.

For randomized controlled trials, the Cochrane Risk of Bias 2 (RoB 2) tool was used. The assessment focused on the adequacy of randomization, deviations from the intended intervention, completeness of outcome data, outcome measurement, and the potential for selective reporting. Any disagreements between reviewers were resolved through discussion and consensus. Because comparability between TOLAC and ERCD groups is particularly important in VBAC research, overall risk-of-bias judgments were based not only on the total Newcastle–Ottawa Scale score but also on domain-specific considerations. Particular attention was paid to the comparability domain and the adequacy of adjustment for clinically relevant confounders.

### 2.6. Data Synthesis and Statistical Analysis

Pooled risk ratios (RRs) with 95% confidence intervals (CIs) were calculated for dichotomous maternal and neonatal outcomes when comparative data were available, whereas pooled proportions with 95% CIs were used to estimate VBAC success rates. The pooled VBAC success rate was derived by combining the proportion of successful vaginal births among women undergoing TOLAC across all eligible studies. Data on maternal and neonatal outcomes were extracted from all eligible studies. Given the variability in outcome reporting across studies, pooled estimates were based on the most complete data available for each outcome. Studies without a direct ERCD comparison group contributed to the pooled VBAC success analysis and narrative synthesis, whereas comparative analyses were restricted to studies reporting relevant outcome data. Because clinical and methodological heterogeneity was anticipated, random-effects models were used throughout the analyses. Between-study variance was estimated using the DerSimonian–Laird method. Statistical analyses were performed using IBM SPSS Statistics for Windows, version 29.0 (IBM Corp., Armonk, NY, USA). No transformations, including logit or Freeman–Tukey double arcsine transformations, were applied before pooling proportions. Zero-event outcomes were assessed separately for each analysis. Studies with zero events in both groups were considered non-informative for estimating relative effect measures and were not included in the corresponding pooled analyses. Continuity corrections were applied only when required for the estimation of effect measures. Statistical heterogeneity was assessed using Cochran’s Q test and the I^2^ statistic, with values of 25%, 50%, and 75% indicating low, moderate, and high heterogeneity, respectively. Exploratory subgroup analyses of VBAC success were performed according to geographic region and study quality. Forest plots were generated for outcomes with at least two directly extractable informative studies, and the number of studies displayed in each forest plot was matched to the corresponding entry in Table 4. Publication bias was assessed using funnel plot inspection and Egger’s regression test when at least 10 informative studies were available. Sensitivity analyses were performed, where applicable, by considering the revised risk-of-bias classification and by excluding studies with a high risk of bias or extreme effect sizes to evaluate the robustness and stability of the pooled estimates.

### 2.7. Certainty of Evidence Assessment

A detailed GRADE Evidence Profile and Summary of Findings table is provided in Supplementary Table S4.

### 2.8. Protocol Deviations

Minor deviations from the original PROSPERO registration occurred during the conduct of the review. Specifically, the final literature search was updated through January 2026 to ensure inclusion of the most recent evidence. In addition, the protocol publication associated with the included randomized controlled trial was retained solely to supplement methodological information. It was not considered an independent source of outcome data and was not formally assessed using the RoB 2 tool. These modifications did not alter the primary objectives or overall methodological approach of the review. During the preparation of this manuscript, the authors used ChatGPT 4 and DeepSeek for grammar checking, spelling correction, and improving written language structure. The authors have reviewed and edited the output and take full responsibility for the content of this publication.

## 3. Results

### 3.1. Study Selection and Characteristics

The included studies comprised one completed randomized controlled trial and 30 observational studies (26 retrospective and four prospective), published between 2010 and 2025 (Table 1). An additional protocol publication associated with the randomized trial was retained solely to supplement methodological information and did not contribute outcome data to the quantitative synthesis. Several included studies focused primarily on VBAC prediction, determinants of successful TOLAC, or descriptive clinical experience rather than direct comparisons between TOLAC and ERCD. Consequently, their contribution was limited to pooled VBAC success estimates and narrative synthesis. Selected cohorts of women with two previous cesarean deliveries (VBA2C) were retained because they met the predefined eligibility criteria and consisted predominantly of women with previous low-transverse uterine incisions.

**Table 1:** Characteristics of Included Studies. Note: One protocol publication associated with the included randomized controlled trial was retained solely to provide methodological information and was not considered an independent source of outcome data.

Author (Year)	Study Design	Country	TOLAC Group (n)	ERCD Group (n)	VBAC Success (%)	Primary Outcome/Study Focus	Neonatal Outcomes
Li et al. (2019) [26]	Retrospective cohort	China	2006	NR	84.0	VBAC success prediction	NICU admission, low Apgar score
Kiwan & Al Qahtani (2018) [27]	Retrospective cohort	Saudi Arabia	567	NR	64.0	VBAC success; induction versus spontaneous labor	NR
De Leo et al. (2020) [28]	Retrospective cohort	Italy	46	68	76.1	Maternal outcomes; VBA2C success	Apgar score
Zhang et al. (2020) [29]	Retrospective cohort	China	708	NR	82.8	VBAC prediction	Neonatal asphyxia
Rozen et al. (2011) [30]	Retrospective cohort	Australia	347	NR	72.0	Maternal complications	NR
Vankan et al. (2017) [31]	Retrospective cohort	Netherlands	515	NR	72.0	VBAC practice variation	NR
He et al. (2016) [32]	Retrospective cohort	China	182	NR	70.0	VBAC success	NR
Familiari et al. (2020) [33]	Retrospective cohort	Italy	300	NR	74.7	Maternal outcomes; VBAC predictors	Apgar score, NICU admission
Lazarou et al. (2021) [34]	Retrospective cohort	Germany	348	NR	70.1	VBAC risk factors	NR
Rusavy et al. (2019) [35]	Retrospective cohort	Czech Republic	268	NR	77.0	Labor duration; genital trauma	Apgar score, birth injuries
Tilden et al. (2017) [36]	Retrospective cohort	USA	245	NR	68.0	VBAC success by birth setting	NICU admission, Apgar score
Paymova et al. (2021) [37]	Case–control	Czech Republic	56	56	61.0	Levator ani avulsion	NR
Bayrampour et al. (2021) [38]	Retrospective cohort	Canada	4741	1014	71.4	Maternal outcomes	NICU admission, Apgar score
Bhide et al. (2016) [39]	Prospective cohort	United Kingdom	1463	NR	71.8	VBAC prediction	NR
Eleje et al. (2019) [40]	Prospective cohort	Nigeria	65	NR	33.8	VBAC determinants	NR
Guo et al. (2019) [41]	Retrospective cohort	China	198	NR	71.0	Antenatal assessment	NR
Tessmer-Tuck et al. (2014) [42]	Prospective cohort	USA	599	NR	76.1	VBAC prediction	NR
Seffah & Adu-Bonsaffoh (2014) [43]	Retrospective cohort	Ghana	2472	NR	61.2	VBAC trends	NR
Chen et al. (2022) [44]	Retrospective cohort	Taiwan	43	NR	86.0	VBAC experience	NR
Liu et al. (2025) [45]	Retrospective cohort	China	720	NR	81.4	VBAC prediction	NR
Zhu et al. (2025) [46]	Retrospective cohort	China	326	184	75.0	Labor duration; maternal outcomes	Apgar score, neonatal trauma
D’Souza et al. (2019) [47]	Retrospective cohort	United Kingdom	238	NR	64.0	Obstetric anal sphincter injury	NR
Masoom et al. (2021) [48]	Retrospective cohort	Pakistan	159	NR	56.0	VBAC success predictors	NR
Modzelewski et al. (2019) [49]	Retrospective cohort	Poland	35	92	62.9	Maternal outcomes	Apgar score, NICU admission
Tesfahun et al. (2023) [50]	Prospective cohort	Ethiopia	345	NR	35.1	VBAC determinants	NR
Mariyam et al. (2025) [51]	Retrospective cohort	United Arab Emirates	1308	NR	38.0	Maternal age and VBAC success	NR
Carauleanu et al. (2021) [52]	Case series	Romania	84	NR	66.0	VBAC experience	NR
Fu et al. (2010) [53]	Retrospective cohort	Taiwan	1302	2180	70.0	VBAC rates by maternal origin	NICU admission, Apgar score
Parveen et al. (2022) [54]	Retrospective cohort	India	1324	NR	65.3	VBAC outcome predictors	NR
Lin et al. (2019) [55]	Prospective cohort	China	162	NR	87.0	VBAC prediction model	NR
Homer et al. (2022) [56]	Randomized controlled trial	Australia	216	218	74.0	Continuity of midwifery care	NICU admission, Apgar score

**Figure 2:**
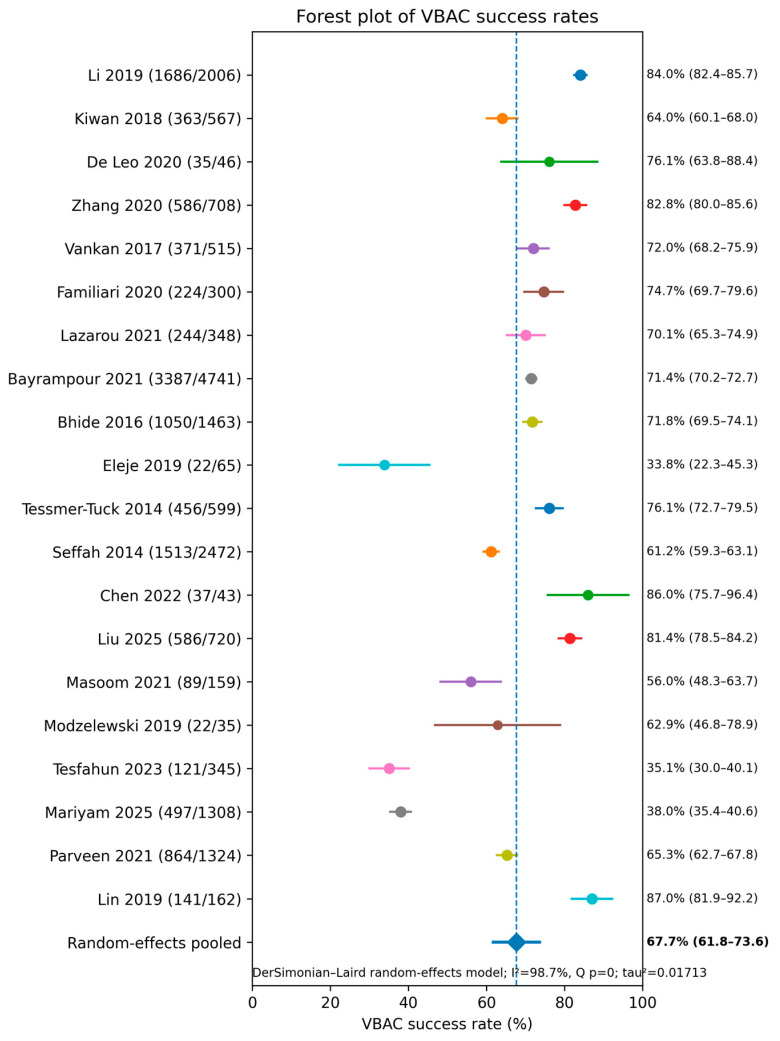
Forest plot of pooled VBAC success rates [26,27,28,29,31,33,34,38,39,40,42,43,44,45,48,49,50,51,54,55].

NR = not reported. Protocol publication was not listed as an independent study in this table because it did not contribute outcome data. Not all included studies had a direct ERCD comparison group; studies without ERCD data contributed only to pooled VBAC success estimates and descriptive synthesis. For studies contributing to the VBAC success meta-analysis, TOLAC denominators and VBAC success percentages were aligned with the extracted study-level data used in Figure 2.

### 3.2. Risk of Bias Assessment

The methodological quality of the included observational studies was generally acceptable but variable. Following a conservative re-assessment using the Newcastle–Ottawa Scale, 17 observational studies were classified as low risk of bias, 12 were classified as moderate risk of bias, and 1 was classified as high risk of bias (Table 2). The most common methodological limitations were retrospective design, single-center setting, limited comparability between groups, and incomplete adjustment for potential confounding factors. Limited comparability between groups and incomplete adjustment for important confounding variables represented the most common reasons for downgrading studies from low to moderate risk of bias.

The randomized evidence was assessed using the Cochrane Risk of Bias 2 (RoB 2) tool. The completed randomized controlled trial by Homer et al. (2022) [57] was judged to have an overall low risk of bias. The accompanying protocol publication by Homer et al. (2013) [57] was retained solely to supplement methodological information and was not considered a source of outcome data. Therefore, it was not formally subjected to RoB 2 assessment. The corresponding assessment is presented in Table 3.

### 3.3. Synthesis of Results

#### 3.3.1. Primary Outcome: Vbac Success Rate

**Figure 3:**
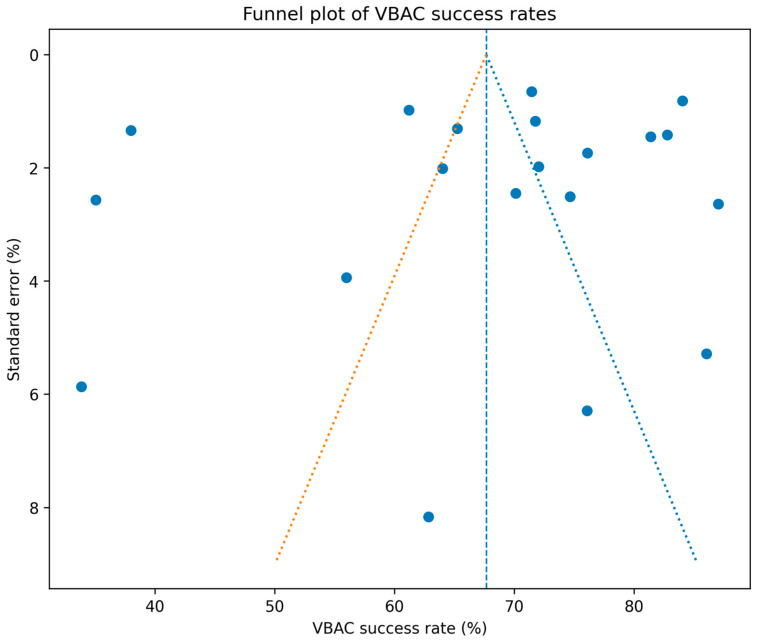
Funnel plot for VBAC success and assessment of publication bias.

**Figure 4:**
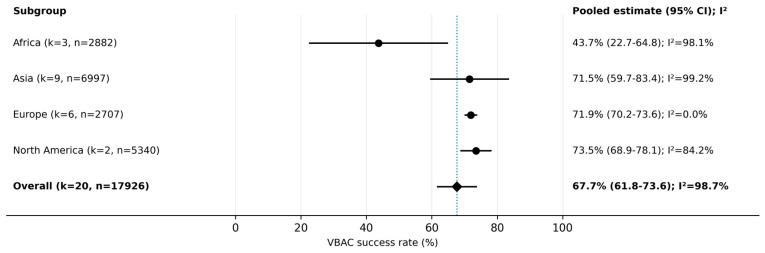
Random-effects subgroup meta-analysis of VBAC success by geographic region.

**Figure 5:**
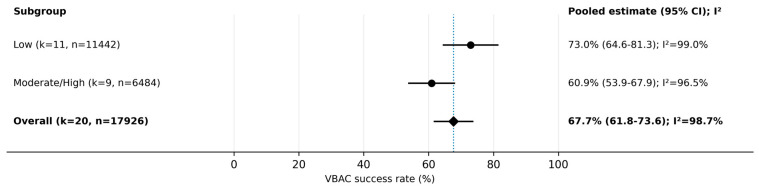
Random-effects subgroup meta-analysis of VBAC success by study quality.

The pooled VBAC success rate across the included studies was 67.7% (95% confidence interval [CI], 61.8–73.6%), with substantial statistical heterogeneity (I^2^ = 98.7%). The distribution of study-specific VBAC success rates and the pooled estimate are presented in Figure 2. Publication bias was assessed using funnel plot inspection and Egger’s regression test, which did not indicate significant small-study effects (Egger test *p* = 0.462; Figure 3). To explore the high between-study heterogeneity, random-effects subgroup meta-analyses were conducted according to geographic region and study quality (Figure 4 and Figure 5).

##### Exploratory Subgroup Analyses of VBAC Success

Exploratory random-effects subgroup meta-analyses were performed to further examine the substantial heterogeneity observed in the pooled VBAC success analysis. When studies were grouped by geographic region, pooled VBAC success rates varied across regions: Africa, 43.7% (95% CI, 22.7–64.8%; I^2^ = 98.1%); Asia, 71.5% (95% CI, 59.7–83.4%; I^2^ = 99.2%); Europe, 71.9% (95% CI, 70.2–73.6%; I^2^ = 0.0%); and North America, 73.5% (95% CI, 68.9–78.1%; I^2^ = 84.2%). The test for subgroup differences was statistically significant (Q = 249.90, df = 3, *p* < 0.001), suggesting that geographic region contributed to between-study variability (Figure 4). When grouped according to study quality, studies at low risk of bias showed a pooled VBAC success rate of 73.0% (95% CI, 64.6–81.3%; I^2^ = 99.0%), whereas studies at moderate or high risk of bias showed a pooled success rate of 60.9% (95% CI, 53.9–67.9%; I^2^ = 96.5%). The test for subgroup differences was also statistically significant (Q = 206.42, df = 1, *p* < 0.001) (Figure 5). These analyses were exploratory and should be interpreted cautiously because heterogeneity remained high within several subgroups.

#### 3.3.2. Maternal Outcomes

**Figure 6:**
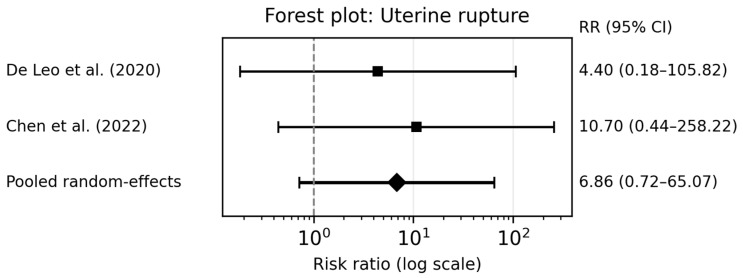
Forest plot of uterine rupture [44,52].

**Figure 7:**
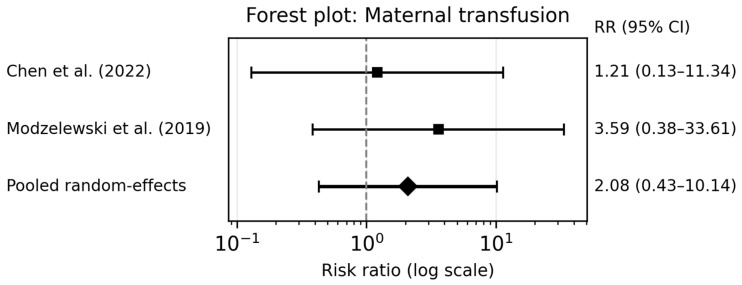
Forest plot of maternal transfusion [44,49].

**Figure 8:**
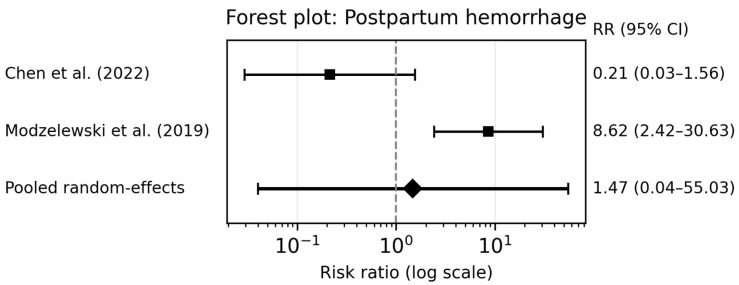
Forest plot of postpartum hemorrhage [44,49].

As summarized in Table 4, uterine rupture was associated with a higher risk among women undergoing TOLAC compared with ERCD (RR 6.86, 95% CI 0.72–65.07), although the estimate was based on two informative comparative studies and was therefore imprecise; the same two studies are displayed in the corresponding forest plot (Figure 6). No statistically significant difference was observed for maternal transfusion, which was also based on two informative studies (RR 2.08, 95% CI 0.43–10.14; Figure 7). Pooled analysis for postpartum hemorrhage included two informative studies and demonstrated substantial heterogeneity (RR 1.47, 95% CI 0.04–55.03; I^2^ = 89.4%; Figure 8), so this result should be interpreted with caution. For hysterectomy and surgical injury, the available comparative data were insufficient to support robust quantitative synthesis.

**Table 4:** Summary of Meta-Analysis Results for Key Comparative Outcomes.

Outcome	Studies Included in Forest Plot/Meta-Analysis (n)	Pooled Risk Ratio (95% CI)	I^2^ (%)	Interpretation
Uterine rupture	2	6.86 (0.72–65.07)	0	Higher risk with TOLAC, but estimate imprecise
Maternal transfusion	2	2.08 (0.43–10.14)	0	No statistically significant difference
Postpartum hemorrhage	2	1.47 (0.04–55.03)	89.4	Considerable heterogeneity; interpret with caution
Perinatal mortality	1	Not pooled	–	Insufficient comparative data
Hysterectomy	0	Not pooled	–	Only double-zero studies available
Surgical injury	1	Not pooled	–	Insufficient comparative data

Abbreviations: RR, risk ratio; CI, confidence interval; I^2^, heterogeneity statistic. Notes: Quantitative synthesis was performed only for outcomes with sufficient directly extractable comparative data. For uterine rupture, maternal transfusion, and postpartum hemorrhage, the number of studies in this table corresponds to the studies displayed in Figure 4, Figure 5 and Figure 6. Studies with zero events in both groups were considered non-informative and were excluded from pooled analyses. Outcomes with limited informative studies were summarized descriptively.

#### 3.3.3. Neonatal Outcomes

Neonatal outcomes were reported inconsistently across the included studies, and the definitions of neonatal morbidity varied considerably. Several studies provided information on neonatal outcomes, including perinatal mortality, neonatal asphyxia, low Apgar scores, and NICU admission; however, only a limited number of studies reported directly extractable comparative event counts for TOLAC and ERCD. Consequently, the available evidence was insufficient to support robust quantitative synthesis for most neonatal outcomes. Although isolated studies suggested an increased risk of adverse neonatal outcomes among women undergoing TOLAC, the absolute event rates remained low, and the limited number of informative studies precluded reliable pooled estimates. Therefore, neonatal outcomes were summarized descriptively. These findings should be interpreted with caution because of heterogeneity in outcome definitions, differences in clinical practice across study settings, and incomplete reporting of comparative neonatal data.

#### 3.3.4. Certainty of Evidence

According to the GRADE framework, the certainty of evidence was low for VBAC success and very low to low for comparative outcomes. Certainty was downgraded because most evidence originated from observational studies, several outcomes were based on a limited number of informative comparative studies, and important heterogeneity or imprecision was present. A detailed GRADE Evidence Profile and Summary of Findings table, including outcome-specific explanations for downgrading decisions and consideration of potential upgrading factors, is provided in Supplementary Table S4.

Sensitivity analyses based on the updated risk-of-bias assessment did not materially alter the interpretation of the main findings. The pooled VBAC success estimate remained characterized by substantial heterogeneity, and the comparative maternal outcomes remained limited by sparse informative studies and wide confidence intervals.

## 4. Discussion

### 4.1. Summary of Principal Findings

The present review suggests that VBAC can be achieved in a substantial proportion of appropriately selected women, although the pooled success rate showed considerable between-study heterogeneity. The strongest and most consistently reported predictors of VBAC success across the included studies were previous vaginal birth, spontaneous onset of labor, and favorable cervical status. Comparative analyses were limited by incomplete reporting of directly extractable TOLAC and ERCD event counts. Among outcomes with sufficient data for quantitative synthesis, uterine rupture showed a higher relative risk among women undergoing TOLAC, although the estimate was imprecise because of sparse events. Maternal transfusion did not differ significantly between groups, while postpartum hemorrhage demonstrated substantial heterogeneity. Neonatal outcomes were reported inconsistently and were therefore summarized descriptively.

The random-effects subgroup meta-analyses provide additional context for the very high heterogeneity observed in VBAC success rates. Geographic region and study quality contributed to between-study variability, although neither factor fully explained the heterogeneity because high I^2^ values persisted within several subgroups. Other clinically important variables, including prior vaginal delivery, induction versus spontaneous labor, and the number of previous cesarean deliveries, were not reported consistently enough to support reliable quantitative subgroup analyses or meta-regression. Therefore, the pooled VBAC success estimate should be interpreted as an overall summary rather than a precise prediction applicable to all clinical settings. Differences in healthcare systems, institutional protocols, access to emergency cesarean delivery, and patient selection criteria likely also contributed to the observed heterogeneity.

### 4.2. Comparison with Existing Literature

Our findings are broadly consistent with current international recommendations and previously published evidence regarding trial of labor after cesarean. Recent FIGO good practice recommendations emphasize that VBAC should be considered for appropriately selected women in settings with adequate institutional support and immediate access to emergency obstetric care. Similarly, the American College of Obstetricians and Gynecologists (ACOG) recommends that most women with one previous low-transverse cesarean delivery should be offered counselling regarding TOLAC, with individualized assessment and shared decision-making representing essential components of clinical care.

The present results are also in agreement with previous systematic reviews and large population-based studies. Guise et al. and the more recent review by Fitzpatrick et al. reported that successful VBAC is associated with lower maternal morbidity and avoidance of complications related to repeated cesarean delivery, whereas uterine rupture remains the most important safety concern associated with TOLAC. Although the relative risk of uterine rupture may be increased, the absolute risk remains low, and contemporary recommendations continue to support careful candidate selection rather than routine repeat cesarean delivery for all women with a previous cesarean section.

The pooled VBAC success rate observed in our review is comparable to those reported in earlier reviews and guideline documents. Nevertheless, substantial heterogeneity was observed across studies. Our exploratory subgroup analyses suggest that differences in geographic region and study quality may partly contribute to this variability, although other clinically important factors, including previous vaginal delivery, induction of labor, and the number of prior cesarean deliveries, were not reported consistently enough to permit reliable quantitative analyses.

Importantly, our review also highlights persistent limitations in the current evidence base. Most available studies were observational, outcome definitions varied considerably, and directly extractable comparative data were frequently unavailable. Consequently, the certainty of evidence according to the GRADE framework was predominantly low to very low for several outcomes. These limitations should be considered when interpreting the present findings and underline the need for more standardized outcome reporting and higher-quality comparative studies to strengthen future evidence.

### 4.3. Clinical Implications and Practice Recommendations

The review findings have important clinical implications, including the integration of individual risk profiles, patient values, and risk tolerance into counselling strategies, as well as optimization of candidate selection through prioritization of TOLAC for patients with a higher probability of successful VBAC and a lower risk of complications. Furthermore, the findings highlight the need for appropriate institutional preparedness, including established TOLAC protocols and the availability of emergency cesarean delivery. Finally, induction and augmentation strategies should be individualized and applied cautiously, particularly in women with additional risk factors for uterine rupture. Continuous fetal monitoring should be considered during TOLAC because abnormal fetal heart rate patterns may represent the earliest sign of uterine rupture, although they are not specific to this complication. Other intrapartum factors, including cord compression and nuchal cord, may also contribute to non-reassuring fetal heart rate tracings and transient neonatal depression. A recent retrospective cohort study reported a modest increase in cesarean delivery for non-reassuring fetal heart rate patterns and lower 1-min Apgar scores in the presence of a nuchal cord, without significant differences in umbilical cord blood gases or severe neonatal morbidity [58]. Therefore, continuous fetal surveillance during TOLAC is important not only for early detection of uterine rupture but also for timely recognition of other intrapartum conditions affecting fetal well-being.

### 4.4. Strengths and Limitations

An important strength of this review is the comprehensive search strategy, which captured a large and diverse body of evidence and enhances the generalizability of the findings across different clinical settings. Nevertheless, several limitations should be acknowledged. Most included studies were retrospective observational cohorts and were therefore inherently susceptible to selection bias, residual confounding, and incomplete adjustment for clinically relevant variables. Considerable heterogeneity was also observed across studies with respect to patient characteristics, eligibility criteria for TOLAC, clinical management protocols, and outcome definitions. Furthermore, not all studies reported the same maternal and neonatal outcomes, and directly extractable comparative event data were unavailable for several outcomes. As a result, some pooled estimates were based on a limited number of informative comparative studies, leading to imprecise effect estimates and wide confidence intervals, while other outcomes could only be summarized descriptively. Another limitation relates to the nature of the available evidence. Several included studies focused primarily on predicting VBAC success or identifying factors associated with successful TOLAC rather than directly comparing TOLAC with ERCD. Although these studies contributed valuable information regarding predictors of successful vaginal birth, they were not designed to provide robust comparative outcome data. In addition, formal assessment of publication bias was feasible only for the VBAC success analysis because the number of informative studies available for most secondary outcomes was insufficient for reliable funnel plot construction and statistical testing.

The methodological quality of the included studies was variable. Although most studies were classified as having low or moderate risk of bias after reassessment, several retrospective single-center studies lacked adequate adjustment for important confounding factors. Furthermore, substantial variation existed among healthcare systems and clinical practices across the countries represented in the review. Differences in patient selection for TOLAC, labor induction and augmentation protocols, availability of continuous fetal monitoring, access to emergency cesarean delivery, and neonatal care policies may have influenced both maternal and neonatal outcomes and likely contributed to the heterogeneity observed across analyses. Therefore, the pooled estimates should be interpreted within the context of local clinical practice, institutional resources, and individual patient characteristics rather than being considered universally applicable.

Finally, a small number of descriptive studies and selected cohorts of women with two previous cesarean deliveries were included because they met the predefined eligibility criteria. These studies contributed only to the pooled VBAC success analysis and narrative synthesis and should not be interpreted as equivalent to comparative studies evaluating TOLAC versus ERCD.

## 5. Conclusions

This systematic review and meta-analysis suggests that vaginal birth after cesarean (VBAC) may be a reasonable option for carefully selected women when supported by appropriate institutional resources and access to emergency obstetric care. Previous vaginal birth, spontaneous onset of labor, younger maternal age, and favorable cervical characteristics were consistently associated with a greater likelihood of successful VBAC. However, the available evidence remains limited by the predominance of observational studies, substantial heterogeneity across study populations and clinical settings, and the low to very low certainty of evidence for several key outcomes. As a result, the pooled estimates should be interpreted cautiously and should not be considered universally applicable to all healthcare settings or patient populations. Although uterine rupture remains the principal safety concern associated with TOLAC, absolute event rates were low, and comparative analyses for many maternal and neonatal outcomes were restricted by sparse data and imprecise estimates. Therefore, decisions regarding mode of birth after a previous cesarean delivery should be guided by individualized risk assessment, informed counselling, patient preferences, and shared decision-making. Future research should prioritize standardized outcome definitions, consistent reporting of maternal and neonatal outcomes, and high-quality comparative studies to improve the reliability, precision, and certainty of the evidence supporting clinical practice.

## Figures and Tables

**Table 2:** Newcastle–Ottawa Scale (NOS) Assessment of Included Observational Studies.

Ref	Study	S	C	O	NOS	Risk
[26]	Li et al., 2019	4	1	3	8	Low
[27]	Kiwan & Al Qahtani, 2018	4	0	2	6	Moderate
[28]	De Leo et al., 2020	3	0	3	6	Moderate
[29]	Zhang et al., 2020	4	1	3	8	Low
[30]	Rozen et al., 2011	4	1	3	8	Low
[31]	Vankan et al., 2017	4	1	3	8	Low
[32]	He et al., 2016	3	1	2	6	Moderate
[33]	Familiari et al., 2020	4	1	3	8	Low
[34]	Lazarou et al., 2021	4	1	3	8	Low
[35]	Rusavy et al., 2019	4	1	2	7	Low
[36]	Tilden et al., 2017	4	2	3	9	Low
[37]	Paymova et al., 2021	3	1	2	6	Moderate
[38]	Bayrampour et al., 2021	4	2	3	9	Low
[39]	Bhide et al., 2016	3	1	2	6	Moderate
[40]	Eleje et al., 2019	3	1	2	6	Moderate
[41]	Guo et al., 2019	4	1	2	7	Low
[42]	Tessmer-Tuck et al., 2014	4	2	3	9	Low
[43]	Seffah & Adu-Bonsaffoh, 2014	3	0	2	5	Moderate
[44]	Chen et al., 2022	3	1	2	6	Moderate
[45]	Liu et al., 2025	4	2	3	9	Low
[46]	Zhu et al., 2025	3	1	2	6	Moderate
[47]	D’Souza et al., 2019	4	2	3	9	Low
[48]	Masoom et al., 2021	3	0	2	5	Moderate
[49]	Modzelewski et al., 2019	4	1	3	8	Low
[50]	Tesfahun et al., 2023	3	1	2	6	Moderate
[51]	Mariyam et al., 2025	4	2	3	9	Low
[52]	Carauleanu et al., 2021	2	0	2	4	High
[53]	Fu et al., 2010	4	2	3	9	Low
[54]	Parveen et al., 2022	3	1	2	6	Moderate
[55]	Lin et al., 2019	4	2	3	9	Low

Abbreviations: S = Selection; C = Comparability; O = Outcome; NOS = Newcastle–Ottawa Scale. Risk-of-bias classification: Low risk (7–9 points), Moderate risk (5–6 points), High risk (≤4 points).

**Table 3:** Risk of Bias Assessment of Randomized Controlled Evidence Using the Cochrane RoB 2 Tool.

Ref	Study	Randomization Process	Deviations from Intended Interventions	Missing Outcome Data	Measurement of Outcome	Selection of Reported Result	Overall Risk of Bias
[56]	Homer et al., 2022	Low	Some concerns	Low	Low	Low	Low

Abbreviations: RoB 2, Cochrane Risk of Bias 2 tool. Judgment categories: Low risk, Some concerns, High risk. Note: The protocol publication by Homer et al. (2013) [57] was included solely to provide methodological information and did not contribute outcome data to the quantitative synthesis. Therefore, it was not formally assessed using the Cochrane Risk of Bias 2 tool.

## Data Availability

The extracted study-level datasets used for the meta-analyses, including VBAC success data and comparative outcome data where available, are available from the corresponding author upon reasonable request. All source data were obtained from previously published studies.

## Supplementary Materials

The following supporting information can be downloaded at: https://www.mdpi.com/article/10.3390/medicina62071286/s1, Table S1: Classification of Included Studies According to Their Contribution to the Review; Table S2: Complete Search Strategies; Table S3: PRISMA 2020 Checklist; Table S4: GRADE Evidence Profile and Summary of Findings.
