# Supplementary material for: Trial of Labor After Cesarean and Vaginal Birth After Cesarean: A Systematic Review and Meta-Analysis of Maternal and Neonatal Outcomes
Source: Medicina (Kaunas). 2026 Jul 3;62(7):1286. doi: 10.3390/medicina62071286 (PMC13413987; doi:10.3390/medicina62071286)
Supplement: Supplementary file 1 [file medicina-62-01286-s001.zip › medicina-4379766-supplementary.pdf]

Supplementary Table S1. Classification of Included Studies According to Their Contribution to the Review

Study	Main objective	Comparative TOLAC vs ERCD data	Included in pooled VBAC success analysis	Included in comparative meta-analysis	Narrative synthesis	Prediction analysis
Li et al. (2019)	VBAC prediction model	No	Yes	No	Yes	Yes
Kiwan & Al Qahtani (2018)	Determinants of VBAC success	No	Yes	No	Yes	Yes
De Leo et al. (2020)	Comparative maternal outcomes	Yes	Yes	Yes	Yes	No
Zhang et al. (2020)	VBAC prediction model	No	Yes	No	Yes	Yes
Rozen et al. (2011)	Maternal complications	No	Yes	No	Yes	No
Vankan et al. (2017)	Practice variation	No	Yes	No	Yes	No
He et al. (2016)	VBAC success	No	Yes	No	Yes	No
Familiari et al. (2020)	Maternal outcomes and predictors	No	Yes	No	Yes	Yes
Lazarou et al. (2021)	Risk factors for VBAC	No	Yes	No	Yes	Yes
Rusavy et al. (2019)	Labor characteristics	No	Yes	No	Yes	No
Tilden et al. (2017)	Birth setting and VBAC success	No	Yes	No	Yes	No
Paymova et al. (2021)	Comparative maternal outcomes	Yes	Yes	Yes	Yes	No
Bayrampour et al. (2021)	Comparative maternal outcomes	Yes	Yes	Yes	Yes	No
Bhide et al. (2016)	Prediction model	No	Yes	No	Yes	Yes
Eleje et al. (2019)	Determinants of VBAC success	No	Yes	No	Yes	Yes
Guo et al. (2019)	Antenatal assessment	No	Yes	No	Yes	No
Tessmer-Tuck et al. (2014)	Prediction model	No	Yes	No	Yes	Yes
Seffah & Adu-Bonsaffoh (2014)	Population trends	No	Yes	No	Yes	No
Chen et al. (2022)	VBAC outcomes	Yes	Yes	Yes	Yes	No
Liu et al. (2025)	Prediction model	No	Yes	No	Yes	Yes
Zhu et al. (2025)	Comparative maternal outcomes	Yes	Yes	Yes	Yes	No
D'Souza et al. (2019)	Obstetric anal sphincter injury	No	Yes	No	Yes	No
Masoom et al. (2021)	Predictors of VBAC success	No	Yes	No	Yes	Yes
Modzelewski et al. (2019)	Comparative maternal outcomes	Yes	Yes	Yes	Yes	No

Tesfahun et al. (2023)	Determinants of VBAC success	No	Yes	No	Yes	Yes
Mariyam et al. (2025)	Maternal age and VBAC success	No	Yes	No	Yes	Yes
Carauleanu et al. (2021)	Clinical experience	No	Yes	No	Yes	No
Fu et al. (2010)	Maternal origin and outcomes	Yes	Yes	Yes	Yes	No
Parveen et al. (2022)	Outcome predictors	No	Yes	No	Yes	Yes
Lin et al. (2019)	Prediction model	No	Yes	No	Yes	Yes
Homer et al. (2022)	Randomized controlled trial	Yes	Yes	Yes	Yes	No

Supplementary Table S2 Complete Search Strategies

Database	Complete search strategy	Date range
**PubMed/MEDLINE**	("vaginal birth after cesarean"[Title/Abstract] OR VBAC OR "trial of labor after cesarean" OR TOLAC) AND ("repeat cesarean" OR "elective repeat cesarean delivery" OR ERCD) AND ("maternal outcomes" OR "neonatal outcomes" OR "uterine rupture" OR "postpartum hemorrhage" OR "maternal morbidity")	January 1, 2010 – January 15, 2026
**Embase (Ovid)**	('vaginal birth after cesarean'/exp OR VBAC OR 'trial of labor after cesarean' OR TOLAC) AND ('repeat cesarean section'/exp OR 'elective repeat cesarean delivery' OR ERCD) AND ('maternal outcome'/exp OR 'neonatal outcome'/exp OR 'uterine rupture'/exp OR 'postpartum hemorrhage'/exp OR 'maternal morbidity'/exp)	January 1, 2010 – January 15, 2026
**CENTRAL (Cochrane Central Register of Controlled Trials)**	("vaginal birth after cesarean" OR VBAC OR "trial of labor after cesarean" OR TOLAC) AND ("repeat cesarean" OR "elective repeat cesarean delivery" OR ERCD) AND ("maternal outcomes" OR "neonatal outcomes" OR "uterine rupture" OR "postpartum hemorrhage" OR "maternal morbidity")	January 1, 2010 – January 15, 2026

Supplementary Table S3. PRISMA 2020 Checklist

Section and Topic	Item No.	PRISMA 2020 Checklist Item	Location in Manuscript
Title	1	Identify the report as a systematic review and meta-analysis	Title page
Abstract	2	Structured abstract	Abstract

Rationale	3	Describe rationale for the review	Introduction
Objectives	4	Provide explicit objectives	End of Introduction
Eligibility criteria	5	Specify inclusion and exclusion criteria	Section 2.2
Information sources	6	Specify databases and search dates	Section 2.1
Search strategy	7	Present full search strategies	Section 2.1; Supplementary Table S2
Selection process	8	Specify screening methods	Section 2.3
Data collection process	9	Describe data extraction methods	Section 2.4
Data items	10a	Outcomes sought	Section 2.4
Data items	10b	Other variables collected	Section 2.4
Risk of bias assessment	11	Specify risk-of-bias methods	Section 2.5
Effect measures	12	Specify effect measures	Section 2.6
Synthesis methods	13a	Eligibility for synthesis	Section 2.6
Synthesis methods	13b	Data preparation methods	Section 2.6
Synthesis methods	13c	Presentation methods	Section 2.6
Synthesis methods	13d	Meta-analysis methods	Section 2.6
Synthesis methods	13e	Heterogeneity exploration	Section 2.6; Section 3.3.1.1
Synthesis methods	13f	Sensitivity analyses	Section 2.6; Section 3.3.4
Reporting bias assessment	14	Methods used to assess publication bias	Section 2.6
Certainty assessment	15	Methods used to assess certainty of evidence	Section 2.7
Study selection	16a	Results of search and selection process	Figure 1; Section 3.1
Study selection	16b	Excluded studies and reasons	Figure 1; Supplementary Table S2
Study characteristics	17	Characteristics of included studies	Table 1
Risk of bias in studies	18	Present risk-of-bias assessments	Tables 2 and 3
Results of individual studies	19	Present results of included studies	Table 1
Results of syntheses	20a	Summaries of characteristics	Section 3.3
Results of syntheses	20b	Statistical synthesis results	Figures 2–8; Table 4
Results of syntheses	20c	Heterogeneity investigations	Section 3.3.1.1
Results of syntheses	20d	Sensitivity analyses	Section 3.3.4
Reporting biases	21	Results of publication bias assessment	Figure 3; Section 3.3.1
Certainty of evidence	22	Present certainty assessments	Table 5
Discussion	23a	General interpretation	Section 4.1
Discussion	23b	Limitations of evidence	Section 4.4
Discussion	23c	Limitations of review process	Section 4.4
Discussion	23d	Implications for practice and research	Sections 4.3 and 5
Registration and protocol	24a	Registration information	Section 2
Registration and protocol	24b	Protocol access	Section 2
Registration and protocol	24c	Amendments to protocol	Section 2.8
Support	25	Sources of support	Funding Statement
Competing interests	26	Declaration of interests	Conflicts of Interest
Availability of data	27	Availability of data and materials	Data Availability Statement

Supplementary Table S4. GRADE Evidence Profile and Summary of Findings

Outcome	No. of informative studies	Study design	Risk of bias	Inconsistency	Indirectness	Imprecision	Publication bias	Reasons for downgrading	Upgrading factors considered	Overall certainty
**VBAC success**	31	1 RCT + 30 observational	Serious	Very serious (I^2=98.7%)	Not serious	Not serious	Undetected	Observational evidence and substantial heterogeneity	Large sample size considered; no upgrade applied	⊕⊕◯◯ Low
**Uterine rupture**	2	Observational	Serious	Not serious (I^2=0%)	Not serious	Very serious	Not assessed	Sparse events and very wide confidence intervals	Large RR considered but insufficient for upgrading	⊕◯◯◯ Very low
**Maternal transfusion**	2	Observational	Serious	Not serious (I^2=0%)	Not serious	Very serious	Not assessed	Limited informative studies and imprecision	No upgrading factors identified	⊕◯◯◯ Very low
**Postpartum hemorrhage**	2	Observational	Serious	Serious (I^2=89.4%)	Not serious	Very serious	Not assessed	Substantial heterogeneity and wide confidence intervals	No upgrading factors identified	⊕◯◯◯ Very low
**Perinatal mortality**	1	Observational	Serious	Not assessable	Not serious	Very serious	Not assessed	Single informative study and sparse events	No upgrading factors identified	⊕◯◯◯ Very low
**Hysterectomy / Surgical injury**	0–1	Observational	Serious	Not assessable	Not serious	Very serious	Not assessed	Rare events and insufficient comparative data	No upgrading factors identified	⊕◯◯◯ Very low

Footnotes

Risk of bias: downgraded because most evidence originated from observational studies.

Inconsistency: predefined as important when I^2 exceeded 50%.

Imprecision: downgraded when event numbers were low or confidence intervals were wide.

Publication bias: formally assessed only for VBAC success using funnel plot inspection and Egger's regression test. Secondary outcomes had too few informative studies for reliable assessment.

Upgrading factors: large effect sizes and dose-response relationships were considered according to GRADE guidance; however, none of the outcomes fulfilled criteria warranting upgrading.
